# Characterization and Identification of Natural Antimicrobial Peptides on Different Organisms

**DOI:** 10.3390/ijms21030986

**Published:** 2020-02-02

**Authors:** Chia-Ru Chung, Jhih-Hua Jhong, Zhuo Wang, Siyu Chen, Yu Wan, Jorng-Tzong Horng, Tzong-Yi Lee

**Affiliations:** 1 Department of Computer Science and Information Engineering, National Central University, Taoyuan 32001, Taiwan; jjrchris@g.ncu.edu.tw (C.-R.C.); horng@db.csie.ncu.edu.tw (J.-T.H.); 2 Warshel Institute for Computational Biology, The Chinese University of Hong Kong, Shenzhen 518172, China; zhongzhihua@cuhk.edu.cn (J.-H.J.); wangzhuo@cuhk.edu.cn (Z.W.); 3 School of Life and Health Sciences, The Chinese University of Hong Kong, Shenzhen 518172, China; 117010024@link.cuhk.edu.cn (S.C.); 117010252@link.cuhk.edu.cn (Y.W.); 4 Department of Bioinformatics and Medical Engineering, Asia University, Taichung 41359, Taiwan

**Keywords:** antimicrobial peptides, organisms, sequence analysis, machine learning, feature selection

## Abstract

Because of the rapid development of multidrug resistance, conventional antibiotics cannot kill pathogenic bacteria efficiently. New antibiotic treatments such as antimicrobial peptides (AMPs) can provide a possible solution to the antibiotic-resistance crisis. However, the identification of AMPs using experimental methods is expensive and time-consuming. Meanwhile, few studies use amino acid compositions (AACs) and physicochemical properties with different sequence lengths against different organisms to predict AMPs. Therefore, the major purpose of this study is to identify AMPs on seven categories of organisms, including amphibians, humans, fish, insects, plants, bacteria, and mammals. According to the one-rule attribute evaluation, the selected features were used to construct the predictive models based on the random forest algorithm. Compared to the accuracies of iAMP-2L (a web-server for identifying AMPs and their functional types), ADAM (a database of AMP), and MLAMP (a multi-label AMP classifier), the proposed method yielded higher than 92% in predicting AMPs on each category. Additionally, the sensitivities of the proposed models in the prediction of AMPs of seven organisms were higher than that of all other tools. Furthermore, several physicochemical properties (charge, hydrophobicity, polarity, polarizability, secondary structure, normalized van der Waals volume, and solvent accessibility) of AMPs were investigated according to their sequence lengths. As a result, the proposed method is a practical means to complement the existing tools in the characterization and identification of AMPs in different organisms.

## 1. Introduction

Antimicrobial peptides (AMPs), naturally encoded by genes and usually containing 12–100 amino acids, are the essential components of the innate immune system and can protect the host from viruses and various pathogenic bacteria [1,2]. They are produced by various organisms, including protozoa, bacteria, and animals, and can cause the cell death of microbes by disrupting either their cell membrane or intracellular functions [3]. In recent years, the prevalent use of antibiotics has resulted in the rapid growth of antibiotic-resistant microorganisms that often induce severe infection and pathogenesis. Since antibiotic resistance is a growing phenomenon in contemporary medicine, the low drug-resistance development of AMPs can provide a possible solution [4].

Several studies have been dedicated to the prediction of AMPs, such as AntiBP [5], AntiBP2 [6], CAMP [7], ClassAMP [8], AVPpred [9], AMPER [10], iAMP-2L [11], iAMPred [12], AmPEP [13], and EFC-FCBF [14]. Specifically, the AMP database, namely APD, has collected 123 human host-defense peptides, 220 AMPs from mammals, 1050 active peptides from amphibians, 116 AMPs from fish, 35 reptile peptides, 40 AMPs from birds, 509 AMPs from arthropods, 160 AMPs from chelicerata, 42 AMPs from molluscs, and 6 AMPs from protozoa [15]. PhytAMP currently contains 271 entries of plant AMPs [16]. Moreover, previous studies have shown that there is a difference in amino acid composition (AAC) among different organisms. Cysteine is a major residue in AMPs from plants, probably because of the advantage of disulfide-bonded and defensive-like molecules [17]. In addition to AACs, the physicochemical property, sequence order, and the pattern of terminal residues have also been adopted in AMP prediction [13]. Furthermore, the net charge, isoelectric point, composition, and tendency for secondary structure are related to the activities of AMPs, such as antibacterial, antifungal, and antiviral activities [6,12,18]. 

With the rapid development of high-throughput proteomic technologies in recent years, machine learning (ML) algorithms have been the primary techniques for building up sequence-based classifiers to distinguish between AMPs and non-AMPs [13]. Mishra and Wang used AACs, physicochemical, and structural features to predict AMPs with different activities based on support vector machine (SVM) [17]. Meher et al. proposed the concept of the adoption of physicochemical features as the features used in ML [12]. Bhadra et al. adopted seven physicochemical classes and three distribution features, identifying where the first residue of a given group is located, and where 25%, 50%, 75%, and 100% of occurrences are contained, to differentiate between AMPs and non-AMPs [13]. Specifically, they proposed the concept of using distribution patterns as features. Additionally, there are several online tools available for the prediction of AMPs. i-AMP2L is a two-level multilabel predictor based on pseudo amino acid composition (PseAAC) and the fuzzy K-nearest neighbor (FKNN) algorithm [11]. It can identify an uncharacterized peptide as AMP or non-AMP based on the amino acid composition and physicochemical properties of sequences [11]. ADAM is a database of AMPs and allows users to predict sequences using SVM and hidden Markov models with amino acid composition adopted as the features [19]. DBAASP is an AMP prediction tool developed from SVM and artificial neural network (ANN) that incorporates hydrophobicity, amphipathicity, location of the peptide in relation to membrane, charge density, propensities to disordered structure, and aggregation being the features [20]. MLAMP adopted ML, synthetic minority oversampling technique (SMOTE), AACs, and physicochemical properties to construct a two-level AMP predictor [21]. CAMPR3 is a database that collects sequences, structures, and family-specific signatures of experimentally validated prokaryotic and eukaryotic AMPs [2]. It also provides AMP prediction tools based on random forest (RF), SVM, ANN, and discriminant analysis (DA), which use AACs, secondary structural propensities, and physicochemical properties as features.

Although AMPs are considered as an alternative drug to conventional antibiotics and has become a model for the development of new drugs that can solve the problem of multidrug resistance, using experimental methods to identify AMPs is expensive and time-consuming. Additionally, few studies have used AACs and physicochemical properties with different sequence lengths against different organisms to predict AMPs. In other words, research devoted to investigating the correlations between AACs/physicochemical properties and different sequence lengths on different organisms is scarce. Therefore, the major purpose of this study is to identify AMPs on seven organisms, including amphibians, humans, fish, insects, plants, bacteria, and mammals. Note that AACs, amino acid pairs, and the physicochemical properties (charge, hydrophobicity, polarity, polarizability, secondary structure, normalized van der Waals volume, and solvent accessibility) of each class are the major features that will be considered. After constructing the AMP classifiers for seven organisms, feature selection methods will be adopted to obtain a better understanding of the sequential characteristics of AMPs with respect to the seven categories of organisms. In addition, we will investigate these features on positions of the sequence to explore their relations. 

## 2. Results

### 2.1. Characterization of AMPs

#### 2.1.1. Compositional Characteristics of AMPs

**Figure 1:**
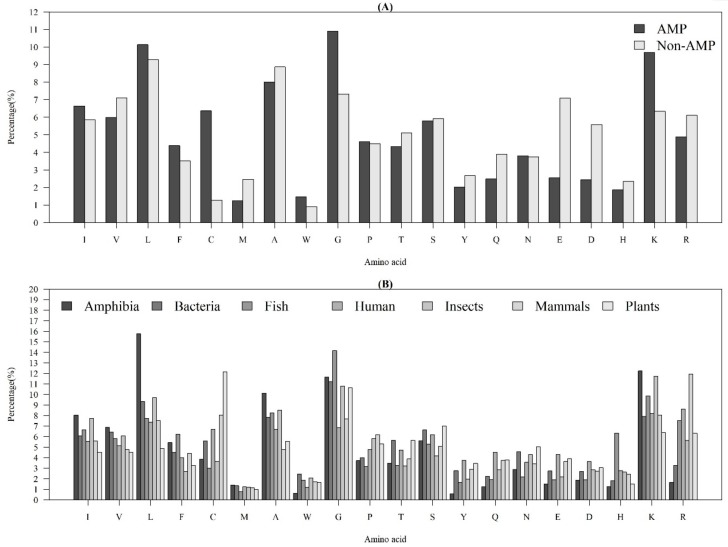
Average AACs of (**A**) AMPs and non-AMPs, and (**B**) AMPs with respect to the seven categories of organisms.

Figure 1A demonstrates the average AACs of AMPs and non-AMPs. Specifically, “L”, “G”, and “K” were abundant amino acids for AMPs, while “L”, “A”, and “G” were abundant amino acids for non-AMPs. Additionally, there was an obvious difference in the composition of “C” (cysteine) between AMPs and non-AMPs. Previous research has indicated that the reason should be due to the dominance from disulfide-bonded and defensing-like molecules [17]. Meanwhile, the composition of “K” (lysine) was different between AMPs and non-AMPs, since the AMP structural cores mainly had positive net charges [22]. The composition of “G” (glycine) of AMPs was higher than the one for non-AMPs. This observation is consistent with that of a previous study, which indicated that the glycine-rich proteins (GRPs) are a group of proteins that occurs in a wide variety of organisms [23].

Figure 1B shows the AACs of AMPs with respect to the seven categories of organisms. There were some obvious differences among these organisms. The AACs related to a hydrophobic property (“C”, “L”, “V”, “I”, “M”, “F”, and “W”) were different among these organisms. Additionally, the composition of “L” (leucine) in Amphibia was much higher than that in the other organisms; the composition of “C” in plants was the highest among the seven categories of organisms; the composition of “K” and “R”, which have positive charges, were higher than that of “E” and “D”, which have negative charges, for each organism. Moreover, the composition of “R” in humans and mammals was higher than that in other organisms. Because of these differences, the AACs were the critical features that differentiated identification of AMPs on different organisms.

#### 2.1.2. Investigation of Physicochemical Properties

**Figure 2:**
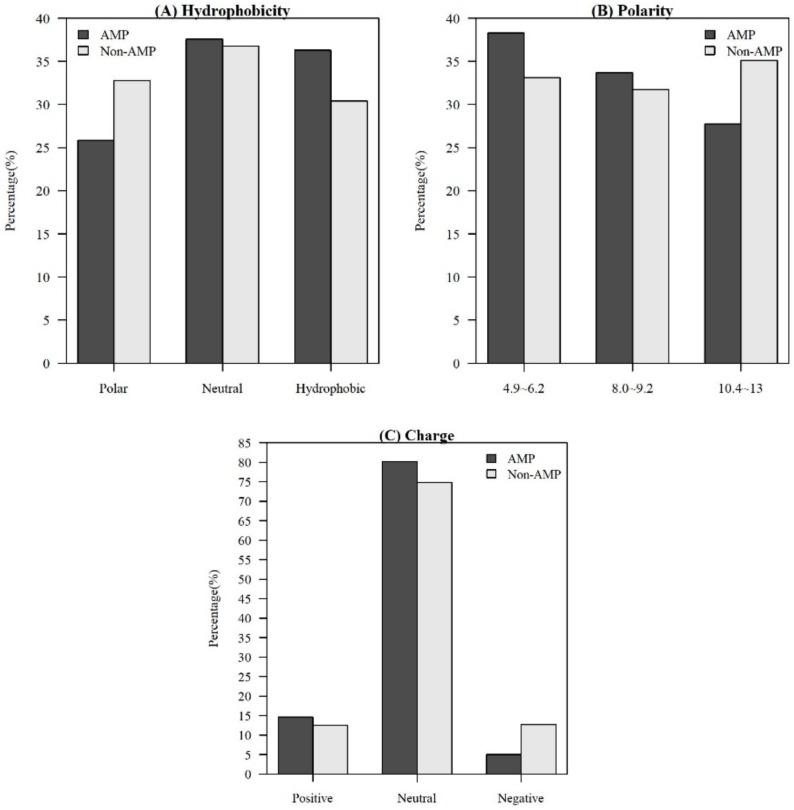
Comparisons of physicochemical properties between AMPs and non-AMPs for (**A**) hydrophobicity, (**B**) polarity, and (**C**) charge.

**Figure A1:**
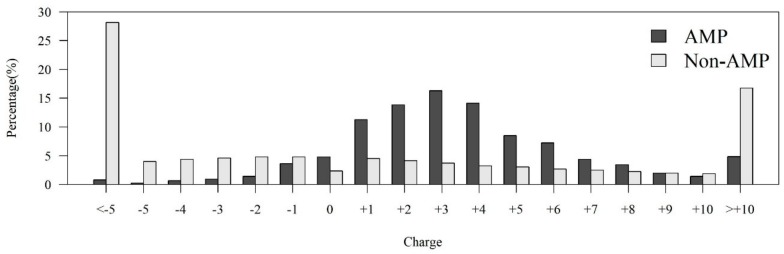
Comparisons of charge distributions between AMPs and non-AMPs.

**Figure A2:**
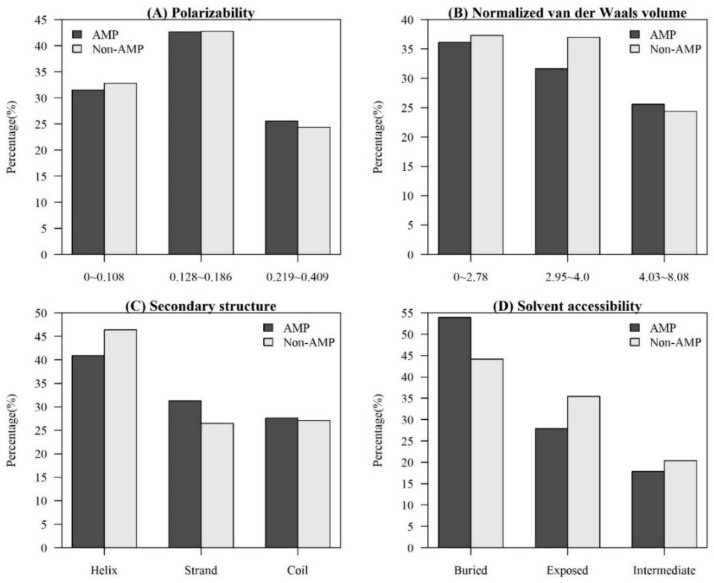
Comparisons of physicochemical properties between AMPs and non-AMPs for (**A**) polarizability, (**B**) normalized van der Waals volume, (**C**) secondary structure, and (**D**) solvent accessibility.

Among the seven physicochemical properties we have collected, it was obvious that there was a significant difference between AMPs and non-AMPs. Figure 2 demonstrates the comparisons of three physicochemical properties between AMPs and non-AMPs. Hydrophobicity was obviously different between AMPs and non-AMPs for the polar class (Figure 2A). The result could be due to the hydrophobic interaction of the hydrophobic face with the lipidic moieties of membranes, which also drives peptide–cell binding [24]. The value of polarity between 4.9 and 6.2 in AMPs was higher than that in non-AMPs (Figure 2B). On the other hand, the value of polarity between 10.4 and 13 in AMPs was lower than that in non-AMPs. The activities of AMPs were found to decrease with an increase in polarity [25]. AMPs tend to be positively charged, which is consistent with previous research where the positive charges were influential in determining AMP activities (Figure 2C) [26]. Appendix A Figure A1 also demonstrates that the AMPs mainly had positive net charges. About half of the AMPs had net charges between +2 and +4, and less than 5% of the AMPs had negative net charges. In addition, the distribution of charges among non-AMPs was different from that of AMPs. Based on these differences in physicochemical properties between AMPs and non-AMPs, we considered these physicochemical features as the important features in the prediction of AMPs. The comparisons of polarizability, normalized van der Waals volume, secondary structure, and solvent accessibility are shown in Appendix A Figure A2. These observations can provide useful information for the construction of AMP classifiers for different classes of organisms and figure out the possible reasons for the high performance of the models.

#### 2.1.3. Physicochemical Properties with Respect to Different Sequence Lengths

**Figure 3:**
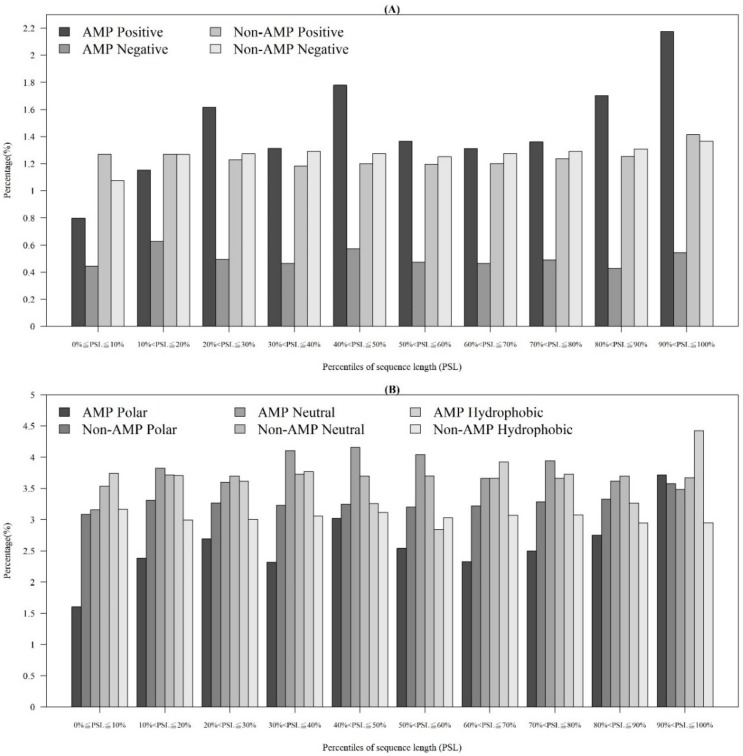
Comparisons of (**A**) charge on different positions of sequence between AMPs and non-AMPs, and (**B**) hydrophobicity at different positions of sequence between AMPs and non-AMPs.

**Figure A3:**
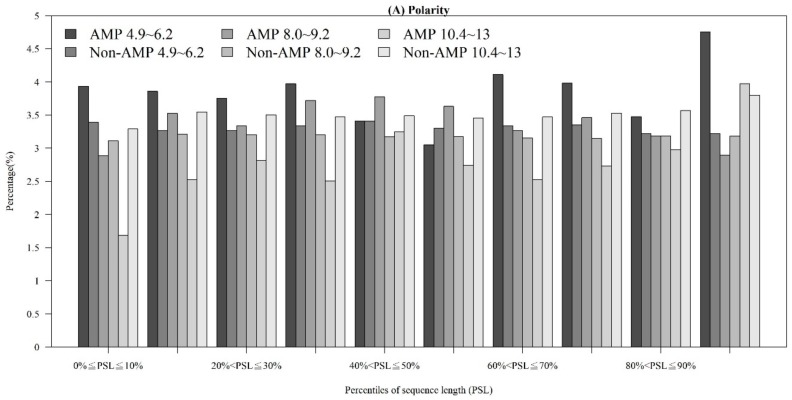

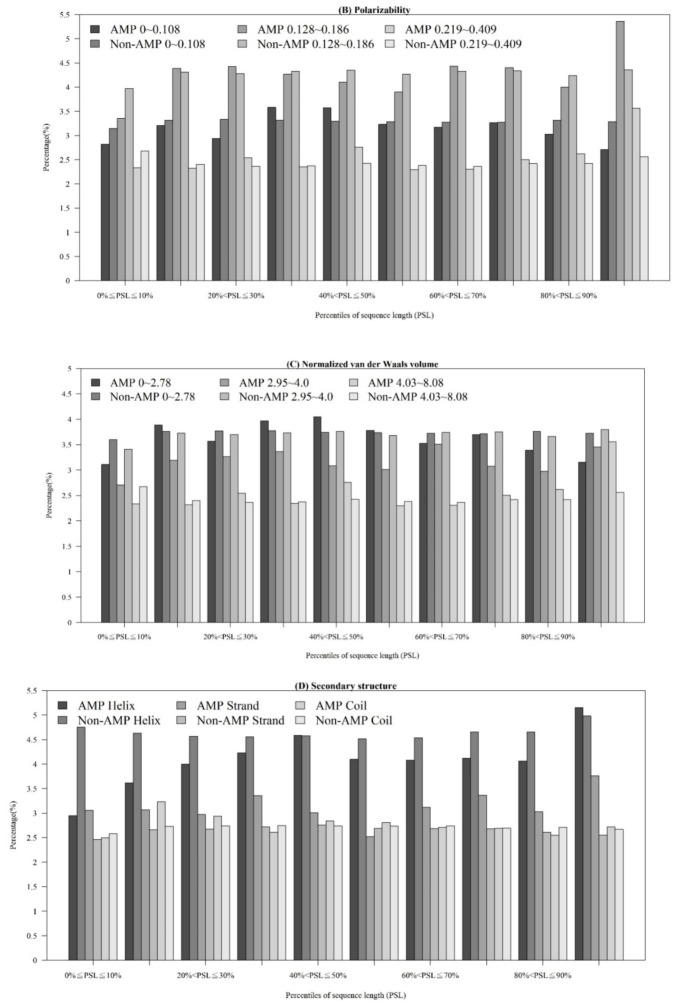

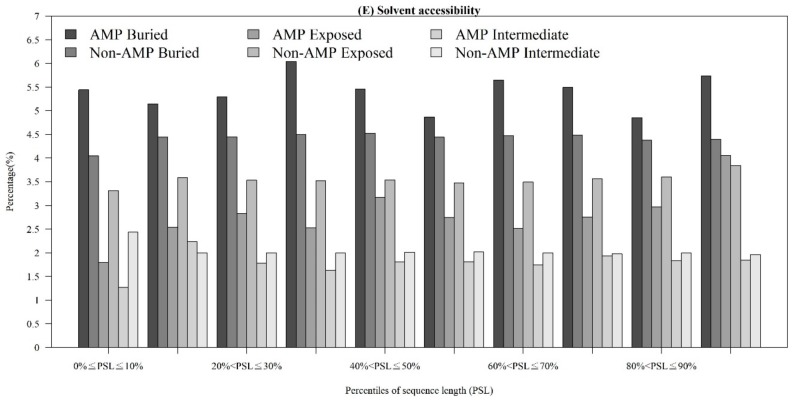
Comparisons of physicochemical properties between AMPs and non-AMPs at different positions (quantiles of sequence length) for (**A**) polarity, (**B**) polarizability, (**C**) normalized van der Waals volume, (**D**) secondary structure, and (**E**) solvent accessibility.

In addition to observing physicochemical properties on AMPs and non-AMPs for different organisms, we also investigated them in different quantiles of sequence length. Figure 3A demonstrates that the majority of AMPs with positive charges were in the 90~100th percentile of sequence length. This is probably because charged amino acids at the tethered C-terminal increased the activity of the peptide. According to these distributions of AMP and non-AMPs, charge is an important feature to predict AMPs. In addition, Figure 3B illustrates the hydrophobicity in different percentiles of sequence length. The majority of AMPs with hydrophobicity were in the 90~100th percentile of sequence length. Previous research has indicated that a more hydrophobic and amphiphilic C-terminal obviously infiltrated into the hydrophobic part of the target cell membrane [27]. Moreover, many physicochemical properties vary among AMPs and different effects on AMP activities such as antibacterial, antifungal, and antiviral activities [22]. Differences can be found in the terminal residue profiles between AMP and non-AMPs. The remaining physicochemical properties also differed at different percentiles of sequence length. The comparisons of polarity, polarizability, normalized van der Waals volume, secondary structure, and solvent accessibility at different percentiles of sequence length are shown in Appendix A Figure A3. These observations can provide some indications on the investigation on the relations between the positions of the sequence and the physicochemical properties of AMPs and non-AMPs.

#### 2.1.4. Physicochemical Properties of AMPs with Respect to Different Categories of Organism

**Figure 4:**
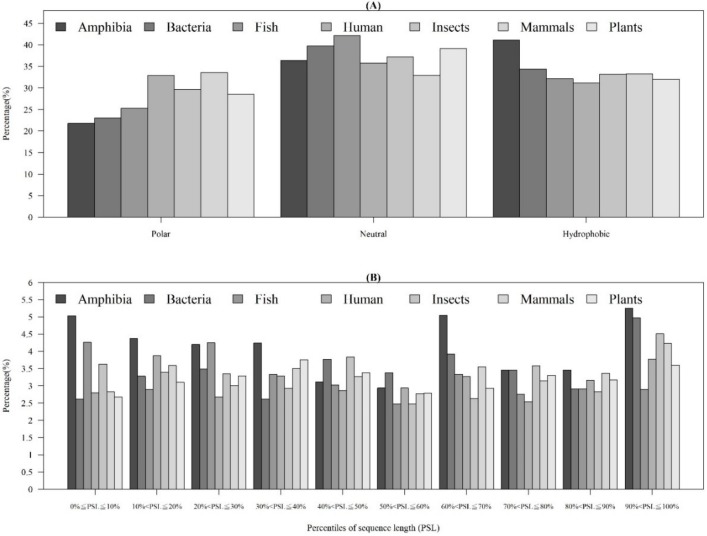
Comparisons of AMP hydrophobicity (**A**) in different categories of organisms and (**B**) at different positions of sequence (percentiles of sequence length) in each category of organism.

**Figure A4:**
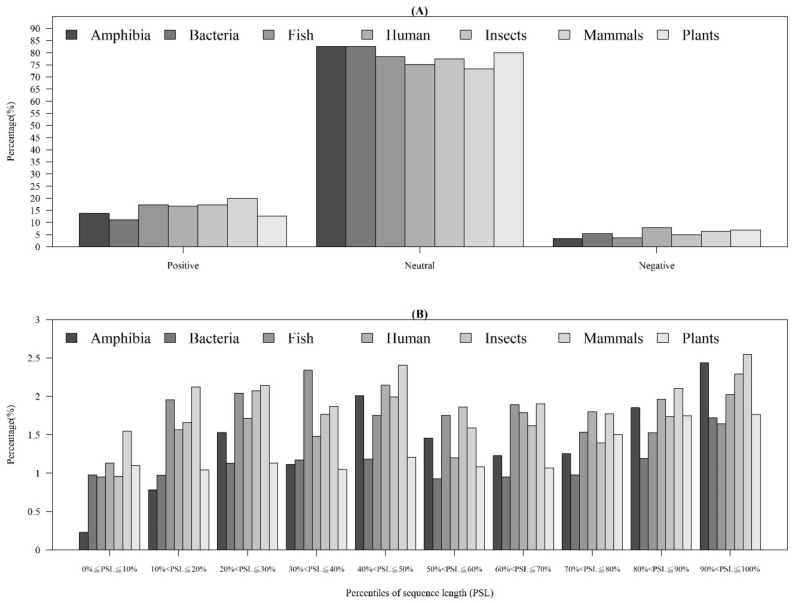
Comparisons of AMP charges (**A**) for different categories of organisms and (**B**) at different positions of sequence (percentiles of sequence length) in each category of organism.

**Figure A5:**
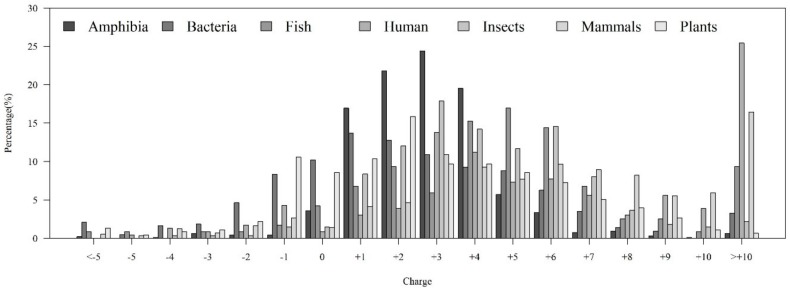
Charge distribution of AMPs from different organisms.

As shown in Table 1, the distribution of AMP sequence lengths among seven categories of organisms indicated that most of AMPs had 20–40 amino acids. Moreover, the number of AMPs with lengths over 100 for human and mammals were much higher than that of other organisms. Figure 4A shows that the AMPs from Amphibia tended to be hydrophobic compared with other organisms. Furthermore, Figure 4B investigates the hydrophobicity of different percentiles of sequence length for each organism. Most of the AMPs from Amphibia, bacteria, insects, and mammals had hydrophobicity in the 90–100th percentile of sequence length. In contrast, the AMPs from humans in the 10–20th and plants in the 30–40th percentiles of sequence length were hydrophobic. Appendix A Figure A4A shows that the percentage of positively charged AMPs was larger than that of the negatively charged AMPs for each category of organism. Appendix A Figure A4B indicates that the positively charged AMPs from Amphibia, insects, and mammals tended to be at larger percentiles of sequence length. Moreover, the distributions of charges in the AMPs from seven organisms are shown in Appendix A Figure A5. We found that the charge distribution was quite different among different organisms. The majority of AMPs from Amphibia had charges between +1 and +4. However, the AMPs from humans and mammals tended to have charges larger than +10 because of the sequence length. Specifically, the number of sequence lengths over 100 from humans and mammals were the largest ones among seven categories of organisms.

### 2.2. The Identification of Important Features

**Figure A6:**
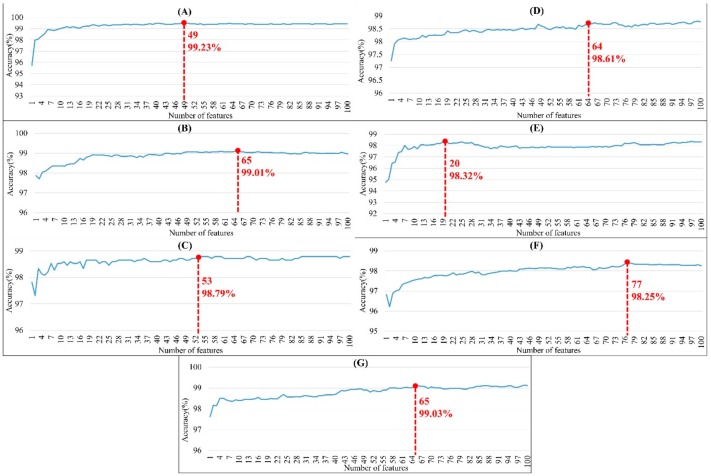
Performance with different numbers of features using forward selection method for (**A**) amphibians, (**B**) bacteria, (**C**) fish, (**D**) humans, (**E**) insects, (**F**) mammals, and (**G**) plants. Note that the red point means the number of features associated with the accuracy for the optimal model.

The order of importance was derived from the random forest algorithm and ranked the features for each category of organism. Appendix A Figure A6 shows that the patterns were accurate when the forward selection method was used to attain the approximate optimal results. These features were included in the prediction model one by one based on the rank order of feature selection. The performance would become better and better when more and more features were put into the prediction model. After a certain number of features were added, the performance curves converged, and further addition of the remaining features only affected the performance slightly. These features were thus selected and adopted in the prediction models, which helped us to reduce the size of the feature set. As shown in Appendix A Figure A6, the final feature sets of Amphibia, bacteria, fish, human, insects, mammals, and plants included the top 49, 65, 53, 64, 20, 77, and 65 features, respectively.

**Figure 5:**
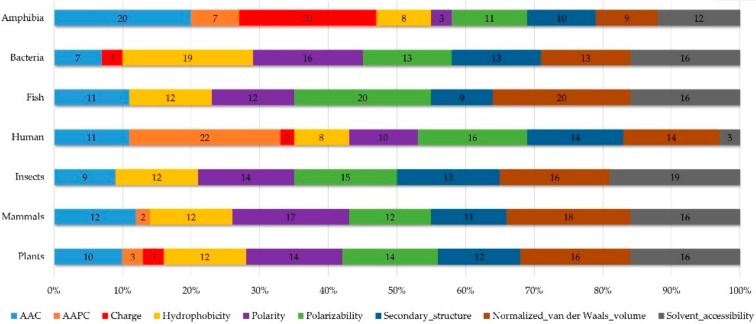
Distribution of features (top 100). Shows the performance of AAC and amino acid pair composition (AAPC), as well as physicochemical composition in different organisms.

**Figure A7:**
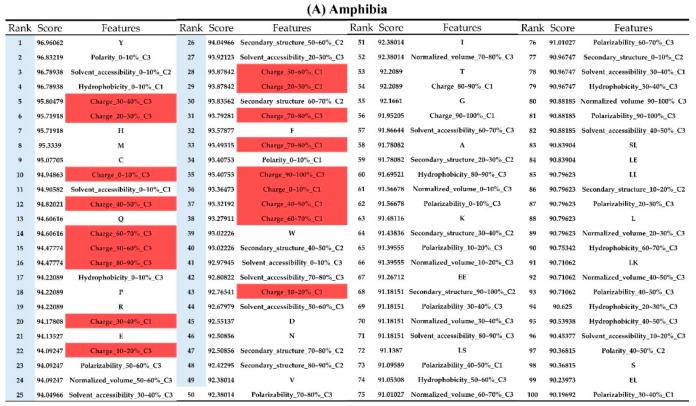

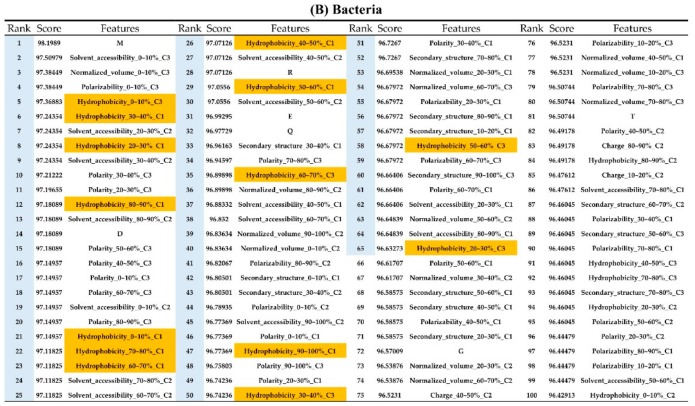

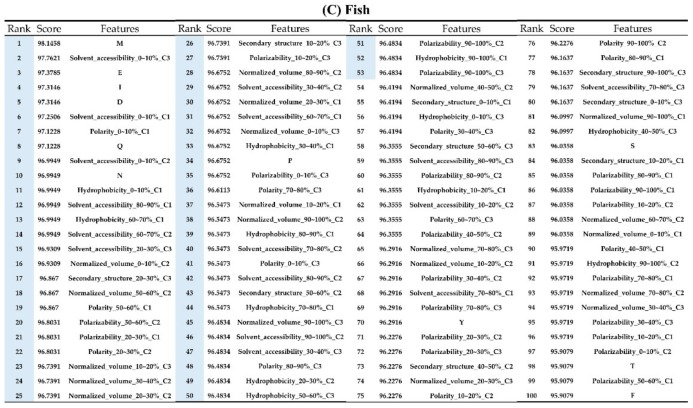

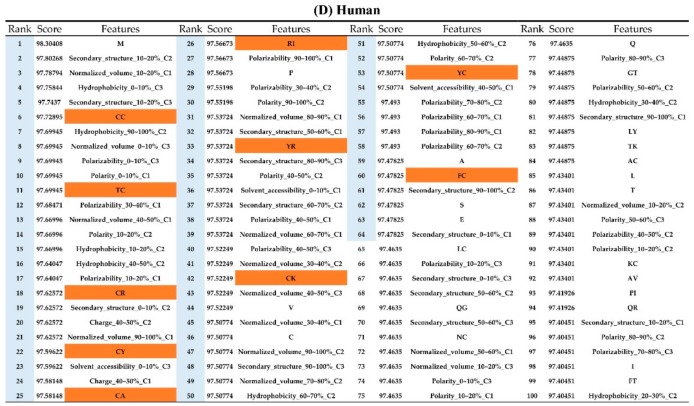

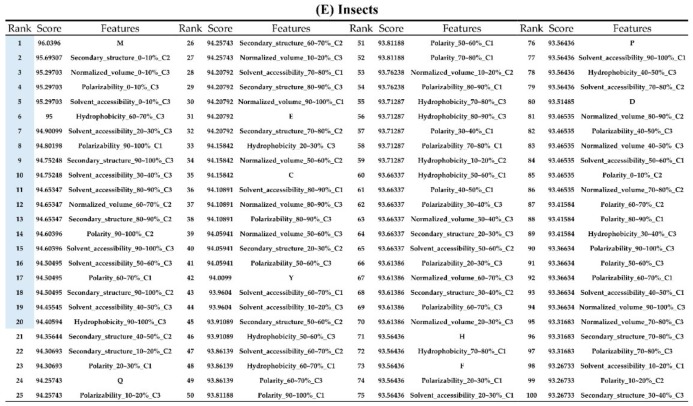

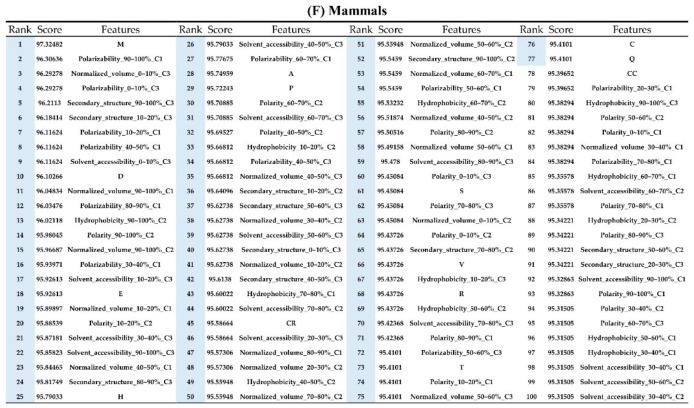

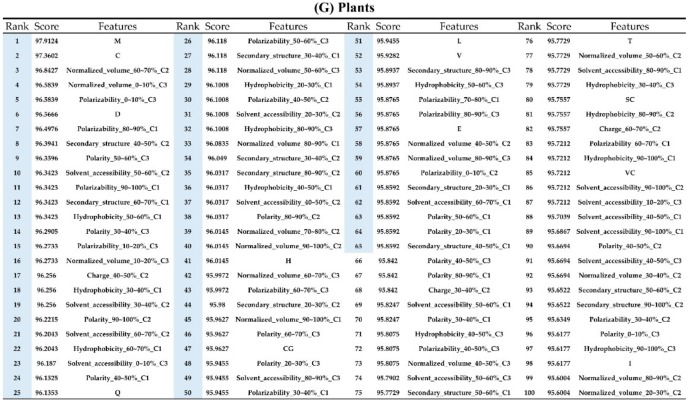
Top 100 features for (**A**) Amphibians, (**B**) bacteria, (**C**) fish, (**D**) humans, (**E**) insects, (**F**) mammals, and (**G**) plants. The rank column with blue background color indicates that the feature was selected from the feature-selection method. The features marked red in (**A**) are related to charge property which is the majority member among the top 100 features for Amphibians. The features marked yellow in (**B**) are associated with the hydrophobicity which is the majority member among the top 100 features for bacteria. The features marked orange in (**D**) are related to AAPC which is the majority member among the top 100 features for human.

**Figure A8:**
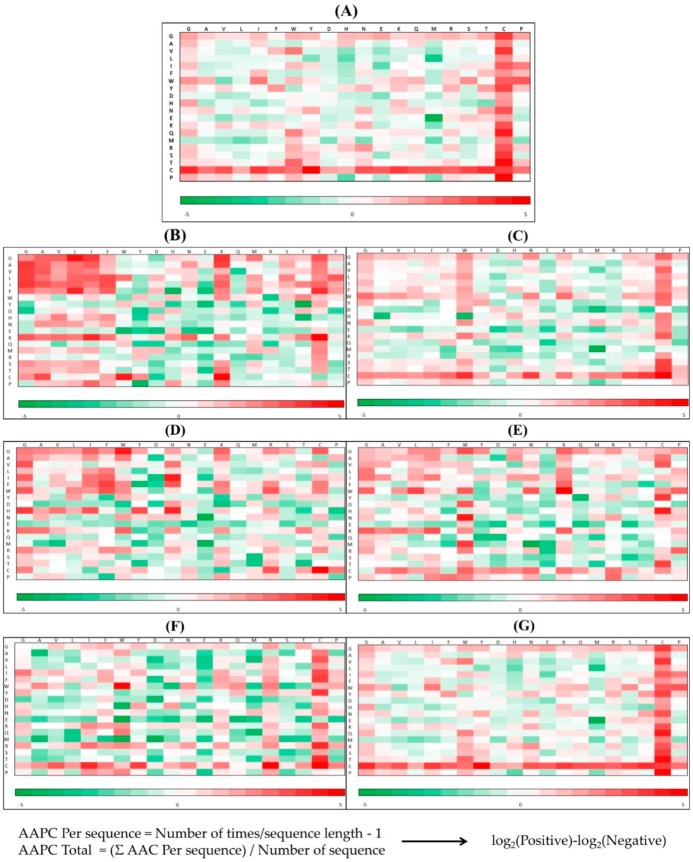
AAPC heatmaps for (**A**) human, (**B**) amphibians, (**C**) bacteria, (**D**) fish, (**E**) insects, (**F**) mammals, and (**G**) plants.

Appendix AFigure A7 demonstrates the details of the top 100 features for each organism after feature selection. These results indicated that the selected features differed among different organisms. As shown in Figure 5, the number of selected features in charge class for Amphibia was much higher than that of the other organisms that could also be found in Appendix A Figure A7A. Therefore, charge is important for the prediction of AMPs of Amphibia. Indeed, a previous study showed that the increase in charge could improve the antimicrobial activity of magainin peptides [28], which are a class of AMPs found in the African clawed frog. In addition, the number of selected features in the hydrophobicity class for bacteria was much higher than that of the other organisms, which could also be found in Appendix A Figure A7B, because the increase in peptide hydrophobicity caused an improvement in antimicrobial activity [29]. The number of selected features in the amino acid pair composition (AAPC) for humans was much higher than that of other organisms, which could also be found in Appendix A Figure A7C. Specifically, the AAPCs of “CC”, “TC”, “CR”, “CY”, and “CA” were ranked in the top 25. Plots of humans are also shown in AAPC heat map (Appendix A Figure A8A), where the color of the regions of “CC”, “TC”, “CR”, “CY”, and “CA” were darker than that of the other amino acid pairs, and these pairs were from human AMPs rather than non-AMPs. The AAPC heat map plots of other organisms are shown in Appendix A Figure A8. Moreover, “C” (cysteine) was the top-ranked feature in plants. Because of the benefit of disulfide-bonded and defensive-like molecules, “C” was the major amino acid residue in AMPs of plants. 

### 2.3. Prediction Performance

The positive training datasets of Amphibians, bacteria, fish, humans, insects, mammals, and plants contained 741, 345, 95, 186, 220, 448, and 364 AMPs, respectively. Accordingly, the negative training dataset contained 1993, 6040, 1469, 6595, 1800, 6919, and 5432 non-AMPs, respectively. The performance of the four classifiers are given in Appendix A Table A1. According to the results, the prediction model can predict not only positive, but also negative data efficiently. Obviously, random forest (RF) was the best classifier for predicting AMPs in these seven categories of organisms. The accuracies of all the models were higher than 93%, and the sensitivities of all categories of organisms were higher than 94%. These results indicate that the used features and RF are efficient for predicting AMPs in each organism.

Furthermore, based on the performance in cross-validation, the RF model was selected to predict the independent test data. The positive test dataset in amphibians, bacteria, fish, humans, insects, mammals, and plants included 185, 86, 23, 46, 54, 111, and 90 AMPs, respectively. Accordingly, the negative test dataset contained 398, 1509, 367, 1648, 450, 1729, and 1358 non-AMPs, respectively. The prediction performance of the independent test is shown in Table 2. All the prediction accuracies of AMPs were above 94%, except that of humans, which was 92.23% but still high. Moreover, the MCCs for all the organisms were larger than 0.650.

### 2.4. Comparison with Other AMP Prediction Tools

**Figure 6:**
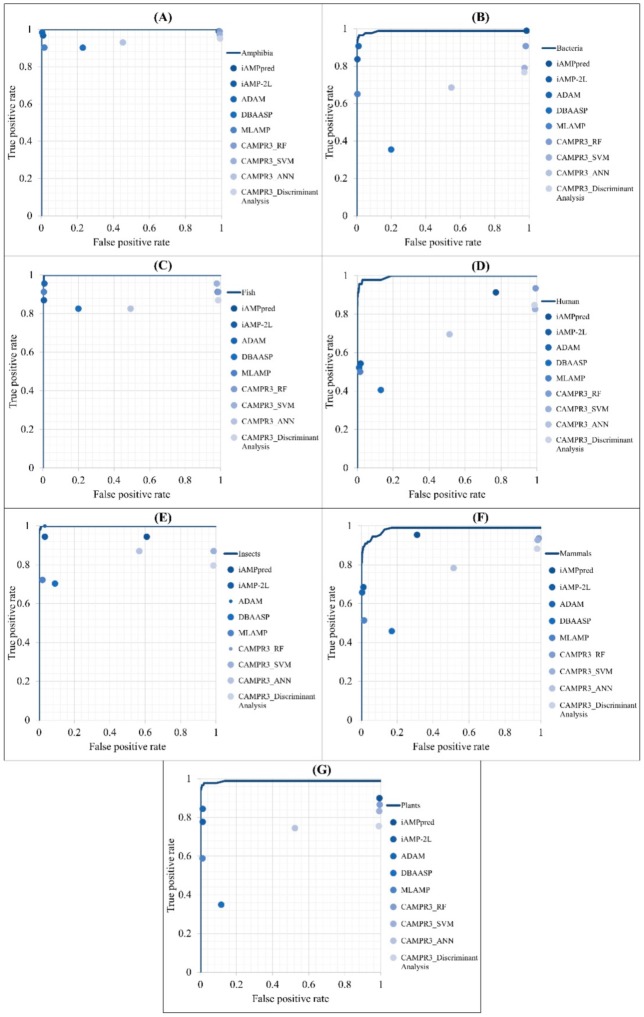
Comparison of ROC curves between our method and other prediction tools in the identification of AMPs on (**A**) Amphibians, (**B**) bacteria, (**C**) fish, (**D**) humans, (**E**) insects, (**F**) mammals, and (**G**) plants.

The performance of predicting the AMPs of different types of organisms was compared with that of other web tools: iAMPpred [12], iAMP-2L [11], ADAM [19], DBAASP [30], MLAMP [31], and CAMPR3 [2]. It should be noted that DBSSAP can only predict peptides with sequence lengths less than 100; therefore, peptides longer than that were removed from our test set to fulfill the requirement. The ROC curves of different models are shown in Figure 6. The comparisons of predicting AMPs for each organism compared with other tools were covered under the ROC curves obtained from our models.

The detailed performance of predicting AMPs in different categories of organisms with the proposed models and other tools are shown in Appendix A Table A2. The accuracies of iAMP-2L, ADAM, MLAMP, and our proposed models were higher than 92% for predicting AMPs from each organism. Additionally, our proposed models reached the highest accuracies when predicting AMPs from insects and plants. Although the accuracies of our proposed models in predicting AMPs in some organisms were not the best, the sensitivities of all our models were the highest. Therefore, the proposed models are efficient in predicting AMPs from different types of organisms.

## 3. Discussion and Conclusions

Because of the rapid development of multidrug resistance, conventional treatment of antibiotics cannot kill pathogenic bacteria efficiently. Additionally, the identification of AMPs using experimental methods is expensive and time-consuming. Computational identification can efficiently and effectively discover candidate peptides as antimicrobial peptides for subsequent experimental assessment, which helps shorten the process of drug discovery [32,33]. In addition, because of the obvious differences in amino acid composition and physicochemical properties (charge, hydrophobicity, etc.) between AMPs and non-AMPs, and the difference in AMPs between different types of organisms, we believe that AMPs can be predicted effectively using these features. Additionally, AMPs from different types of organisms can be differentiated.

This study employed the one-rule attribute evaluation (OneR) method and forward-selection method, reducing the number of features from 630 to 49, 65, 53, 64, 20, 77, and 65, respectively, in amphibians, bacteria, fish, humans, insects, mammals, and plants. Then, four different classification algorithms were used to build predictive models. The performance of the models in five-fold cross-validation indicated that the feature sets were effective in the predictions. Accuracies and AUCs for all organisms were observed to be larger than 93%, which shows that the feature set and random forest method were efficient in predicting AMPs of different organisms. Moreover, we observed the feature sets of the seven types of organisms and found differences among organisms. For instance, electric charge was an important feature in the prediction of AMPs for Amphibia, because the charged residues in Amphibia were the most important features, which had a very high rank among all features of Amphibia. According to these differences in feature sets of the seven categories of organisms, we conclude that AMPs from different types of organisms can be differentiated well.

Furthermore, the performance of the models was compared with that of iAMPpred, iAMP-2L, ADAM, DBAASP, MLAMP, and CAMPR3 using the same testing dataset. The accuracies of iAMP-2L, ADAM, MLAMP, and proposed models were higher than 92% in predicting each organism. In addition, the sensitivity of the proposed models in predicting AMPs of seven organisms were the highest. As a result, the proposed models are believed to complement the existing tools in predicting AMPs and differentiate AMPs on different types of organisms. Last but not least, the proposed methods also lead a promising way to the design of new AMPs, which will enlighten the future of drug development. Accordingly, we believe that the proposed model in preclinical characterization of predicting AMPs will improve the long-term efficiency of AMP drug development.

## 4. Materials and Methods 

### 4.1. Data Collection and Preprocessing

**Figure 7:**
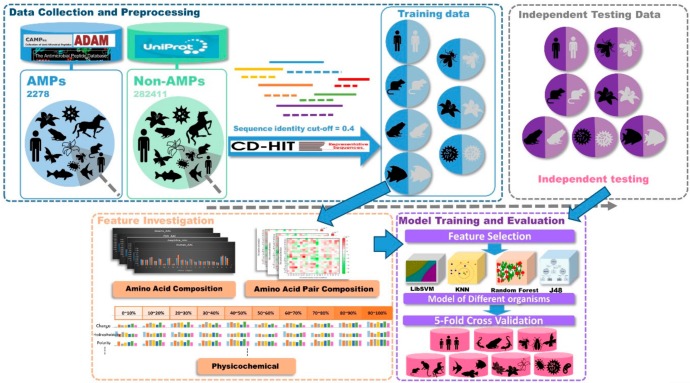
Conceptual framework. This study was divided into three parts: data collection and preprocessing, feature investigation, and model training and evaluation.

This study was divided into three parts as shown in Figure 7, data collection and preprocessing, feature investigation, and model training and evaluation. At first, positive datasets were collected from several databases. Then, AMPs were classified based on the types of organisms they came from. Negative datasets were downloaded from UniProt. After filtering conditions, all the non-AMPs were classified into seven types of organisms. Then, the sequence analysis tool, CD-HIT, was used to remove sequences that were 40% similar to positive dataset sequences in the negative dataset. The independent testing datasets of each organism were generated by drawing 20% of the data from the corresponding organism dataset. The AAC, amino acid pair composition (AAPC), and physicochemical properties in different sequence lengths of data were included in our feature sets. Then, the feature sets of each organism were analyzed by feature-selection methods to dig out the important features. With these selected features, prediction models were designed by four different kinds of algorithms. Finally, the predictive performances were compared after 5-fold cross validation and independent testing.

AMPs are common in nature and have been discovered in almost all forms of life, from single-celled bacteria to multicellular organisms such as animals and plants [17]. In this study, we collected the positive dataset by capturing naturally existing and experimentally validated AMP sequences from different organisms from several databases, CAMP [7], APD [15], ADAM [19], and DRAMP [21]. We collected all the AMPs and deleted the duplicated ones. Then, all the AMPs were classified into seven organisms, which contained 232, 926, 118, 274, 454, 431, and 559 from humans, amphibians, fish, insects, plants, bacteria, and mammals. We followed the data preparation procedure conducted in other studies to generate our negative dataset [11,34]. For the construction of negative data, we extracted protein sequences without the annotations of membrane, toxic, secretory, defensive, antibiotic, anticancer, antiviral, and antifungal properties from UniProt. Unique sequences were collected, which contained 11,275, 3656, 3005, 5225, 24,443, 281,434, and 33,483 non-AMPs from humans, amphibians, fish, insects, plants, bacteria, and mammals. In order to prevent the overestimation of predictive performance in this investigation, the CD-HIT program [35] was applied to remove similar sequences from the training dataset. It would be possible that some negative data were identical to some of the positive data in the training dataset, potentially causing “false positive” or “false negative” predictions. Consequently, CD-HIT was further applied by running CD-HTT-2D across positive and negative training datasets with 100% to 40% sequence identity to solve this problem. In this study, we reduced sequence redundancy of the negative dataset by removing the data with a 40% sequence similarity in all seven negative datasets. Then, for different types of organisms, we compared the sequence similarity between positive and negative datasets, and we removed sequences that were 40% similar to positive dataset sequences in the negative dataset. After filtering, our negative datasets had 8243, 1993, 1836, 2250, 6790, 7549, and 8648 non-AMPs from humans, amphibians, fish, insects, plants, bacteria, and mammals. The independent testing datasets of each organism were generated by separating 20% from the corresponding dataset. A summary of the positive and negative datasets is given in Table 3.

### 4.2. Feature Constructions

AACs were obtained separately for each sequence, so were the ratios of all 20 amino acids. There are 20 amino acids, so this feature set had 20 dimensions. The following is an example of how to obtain AAC from a sequence “AIFIFIRWLLKLGHHGRAPP”. First, we calculated the frequency of the 20 amino acid residues in this sequence. Then, the frequency of isoleucine (I) in this sequence was computed as (3(Number of I)/20(Sequence length)) = 0.15. Finally, the frequency of amino acid residues of this sequence will be calculated as AAC features.

AAPC is the ratio of the occurrences of the amino acids in pairs of two in each sequence. There are 20 amino acids, so this feature was 20 by 20 and equaled 400 dimensions. The same example was adopted to illustrate the determination of AAPC. First, we calculated the number of occurrences for 400 amino acid pairs in this sequence. Then, the frequency of “IF” pairs in the sequence was computed as (3(Number of IF)/19(Sequence length − 1)) = 0.105. Finally, the frequencies of 400 amino acid pairs of this sequence were taken as 400 AAPC features.

Previous studies have organized amino acids into several physicochemical property groups [13,17]. As shown in Appendix A Table A3, seven physicochemical properties were used in the grouping: (1) charge, (2) hydrophobicity, (3) polarity, (4) polarizability, (5) secondary structure, (6) normalized van der Waals volume, and (7) solvent accessibility. For each of these seven physicochemical properties, 20 amino acids were grouped into 3 classes. For example, for the charge property, the 3 classes were positive (K and R), neutral (A, N, C, Q, G, H, I, L, M, F, P, S, T, W, Y and V), and negative (D and E). For each 21 (= 7 × 3) classes, we generated 10 classes based on the percentiles of sequence length, such as 0~ 0, 10~20th, 20~30th, …, and 90~100th percentiles of sequence length. The ratio of each amino acid of each physicochemical property class in each quantile class was calculated. We illustrated these computations with the sample sequence “AALKGCWTKSIPPKPCFGKR” according to the charge property and its three classes, positive, neutral, and negative. First, we split the sequence into 10 partitions, and then we calculated the ratio of the representative amino acids in each partition. The first partition (0–10th quantile) was the sequence “AA”, which did not contain Class 1 and Class 3, but 2 of them were in the Class 2 charge. It means that the number of Class 2 sequences in the 0~10th percentile of sequence length was 2. Finally, the frequency of charge of Class 2 was computed as (2(0–10th percentile contained Class 2)/20(Sequence length)) = 0.1. After these calculations, we could obtain results at ten different positions, seven physicochemical properties of amino acids, three classes for each property, and final 210 (= 7 × 3 × 10) features in total for each sequence. Therefore, each sequence was transformed into 630 features (AAC (20) +AAPC (400) + physicochemical properties in different sequence length (210)).

### 4.3. Model Construction and Feature Selection Methods

In this study, OneR feature selection method was used to select features. This feature selection method can be found in Weka, which was the major analytic tool in this study [36]. OneR is a simple classification algorithm. As its name indicates, it generates a rule to predict the data. A contingency table was constructed for each predictor against the target, and then the best rule with the lowest total error, also named as “one rule”, was selected.

RF is a classifier proposed by Breiman L., who published the ensemble of multiple classifiers based on random feature selection. The main idea about random forests is constructing a multitude of decision trees, and each tree is construct by random sampling of the training data. This machine learning method is considered as an appropriate classifier for processing a large-scale dataset, especially an imbalanced dataset. It corrects the habit of decision trees overfitting their training sets. This method was used in this study and generated by Weka. SVM is a supervised learning model based on associated learning algorithms using regression analysis to classify data [37]. The positive and negative training datasets were used for building a predictive model with the identified support vectors. In this study, a binary classification problem (AMP versus non-AMP) has been considered. The discriminatory ability of an SVM classifier is determined by a hyperplane in a high-dimensional space that can discriminate the AMPs from the non-AMPs. K-nearest neighbor models (KNN) is an instance-based algorithm used in classification. In a binary classification between positive and negative samples, every data point is a vector in a multidimensional feature space with a class label (AMPs or non-AMPs). Users can decide a value k, related to the scale of the subgroup, for prediction. A testing data point without a label was classified using k nearest training samples. In this study, many values of k tried to achieve the best performance. Decision tree (DT) is a tree-like model in which each internal node represents a “test” on an attribute, each branch represents the outcome of the test, and each leaf node represents a class label (positive or negative data) [38]. J48 is a classification model based on constructing a decision tree with the top-down process. The process starts from the test of the root node and follows the appropriate branch based on the test. A tree-like graph with a model of decisions was generated during the prediction. The outcome is the contents of the leaf node, and the conditions along the path is decided by a decision rule. Decision rules can be generated by constructing association rules and can denote temporal or causal relations.

### 4.4. Evaluation Matrics

The predictive models in this study based on machine learning methods have been trained and validated via five-fold cross-validation. The training dataset was divided into five non-overlapping subgroups with approximately equal sizes. In each round, four subgroups were used for training, and one for testing, and then the validation process was repeated five times. Then, the five validation results were combined to generate a single estimation. The performance of the trained models was estimated using sensitivity (*S_n_*), specificity (*S_p_*), accuracy (*A_cc_*), and Matthews correlation coefficient (*MCC*). The definitions are given below.

(1) $$S_{n}=\frac{TP}{TP+FN}$$

(2) $$S_{p}=\frac{TN}{FP+TN}$$

(3) $$A_{cc}=\frac{TP+TN}{TP+TN+FP+FN}$$

(4) $$MCC=\frac{\left(TP\times TN)-(FP\times FN\right)}{\sqrt{\left(TP+FP)\times (TP+FN)\times (FP+TN)\times (TN+FN\right)}}$$

where *TP*, *TN*, *FP*, and *FN* represent the number of true positives, true negatives, false positives, and false negatives, respectively. In this study, to evaluate the performance of the ML models, a ranking list of features was generated by feature selection methods. After using the forward-selection method, the features that resulted in the best performance were used to design the models.

## Figures and Tables

**Table 1:** Distribution of AMP sequence lengths among different organisms on training datasets.

Organisms	Number of Peptides with Length L						
	L ≤ 20	20 < L ≤ 40	40 < L ≤ 60	60 < L ≤ 80	80 < L ≤ 100	100 < L	Total
Amphibia	269	437	28	3	0	4	741
Bacteria	117	111	61	16	13	27	345
Fish	18	54	10	5	3	5	95
Human	11	53	13	26	7	76	186
Insects	67	94	32	12	7	8	220
Mammals	78	180	51	43	11	85	448
Plants	63	153	95	7	14	32	364

**Table 2:** Performance of the models using data from different types of organisms in the independent test.

Organisms	Sensitivity	Specificity	Accuracy	Matthews Correlation Coefficient
Amphibia	100.00%	98.24%	98.80%	0.973
Bacteria	96.51%	96.36%	96.36%	0.746
Fish	100.00%	97.00%	97.18%	0.810
Human	97.83%	92.17%	92.33%	0.482
Insects	100.00%	97.56%	97.82%	0.900
Mammals	92.79%	94.56%	94.46%	0.673
Plants	97.78%	97.94%	97.93%	0.851

**Table 3:** Number of peptides in training and testing datasets among different organisms.

Organisms	Training Dataset		Testing Dataset	
	Positive	Negative	Positive	Negative
Amphibia	741	1595	185	398
Bacteria	345	6040	86	1509
Fish	95	1469	23	367
Human	186	6595	46	1648
Insects	220	1800	54	450
Mammals	448	6919	111	1729
Plants	364	5432	90	1358

## Abbreviations

AMPs	Antimicrobial peptides
AACs	Amino acid compositions
ML	Machine learning
SVM	Support vector machine
PseAAC	Pseudo amino acid composition
FKNN	Fuzzy K-nearest neighbor
ANN	Artificial neural network
SMOTE	Synthetic minority oversampling technique
RF	Random forest
DA	Discriminant analysis
AAPC	Amino acid pair composition
OneR	One rule attribute evaluation
KNN	K-nearest neighbor models
Sn	Sensitivity
Sp	Specificity
Acc	Accuracy
MCC	Matthews correlation coefficient
TP	True positives
TN	True negatives
FP	False positives
FN	False negatives

## Appendix A

**Table A1:** Performance of training datasets for the AMPs derived from different organisms. The optimal models which contain best prediction performance are marked in blue background color. It would be noted that the optimal model was determined as the one with the minimum difference between sensitivity and specificity.

Organisms	Classifier	Sensitivity	Specificity	Accuracy	Matthews Correlation Coefficient
Amphibia	RF	99.19%	99.18%	99.19%	0.981
	DT	97.84%	98.81%	98.50%	0.965
	KNN	96.76%	99.81%	98.84%	0.973
	SVM	98.92%	98.93%	98.93%	0.975
Bacteria	RF	95.94%	96.18%	96.16%	0.735
	DT	86.67%	97.95%	97.34%	0.769
	KNN	73.62%	99.44%	98.04%	0.7959
	SVM	95.94%	95.94%	95.94%	0.725
Fish	RF	96.84%	96.87%	96.87%	0.789
	DT	73.68%	98.43%	96.93%	0.728
	KNN	68.42%	99.52%	97.63%	0.774
	SVM	82.11%	99.86%	98.79%	0.889
Human	RF	94.09%	93.07%	93.10%	0.489
	DT	74.19%	98.15%	97.49%	0.615
	KNN	68.28%	98.94%	98.10%	0.654
	SVM	88.17%	87.82%	87.83%	0.354
Insects	RF	96.36%	96.33%	96.34%	0.838
	DT	91.36%	97.56%	96.88%	0.849
	KNN	85.91%	98.28%	96.93%	0.842
	SVM	95.00%	95.11%	95.10%	0.793
Mammals	RF	94.42%	95.24%	95.19%	0.708
	DT	83.71%	92.60%	92.06%	0.560
	KNN	74.55%	98.92%	97.43%	0.767
	SVM	93.97%	93.97%	93.97%	0.662
Plants	RF	97.53%	97.39%	97.39%	0.822
	DT	88.74%	98.82%	98.19%	0.851
	KNN	80.49%	99.45%	98.26%	0.845
	SVM	96.70%	96.70%	96.70%	0.786

Note. RF = random forest; DT = decision tree; KNN = K-nearest neighbor; SVM = support vector machine.

**Table A2:** Comparisons of independent testing results between our method and other prediction tools in the identification of AMPs on different organisms.

Organisms	Classifier	Sensitivity	Specificity	Accuracy	Matthews Correlation Coefficient
Amphibia	Our method	100.00%	98.24%	98.80%	0.973
	iAMPpred	98.92%	1.51%	32.42%	0.017
	iAMP-2L	96.76%	98.99%	98.28%	0.960
	ADAM	98.38%	99.50%	99.14%	0.980
	DBAASP	90.22%	76.92%	89.34%	0.477
	MLAMP	90.27%	98.24%	95.71%	0.900
	CAMPR3_RF	98.92%	1.01%	32.08%	−0.004
	CAMPR3_SVM	97.30%	1.01%	31.56%	−0.064
	CAMPR3_ANN	92.97%	54.77%	66.90%	0.454
	CAMPR3_DA	95.14%	0.75%	30.70%	−0.135
Bacteria	Our method	96.51%	96.36%	96.36%	0.746
	iAMPpred	84.88%	1.99%	6.46%	−0.183
	iAMP-2L	83.72%	99.54%	98.68%	0.867
	ADAM	90.70%	98.87%	98.43%	0.855
	DBAASP	35.44%	80.00%	57.86%	0.173
	MLAMP	65.12%	99.47%	97.62%	0.743
	CAMPR3_RF	90.70%	1.99%	6.77%	−0.108
	CAMPR3_SVM	79.07%	2.72%	6.83%	−0.218
	CAMPR3_ANN	68.60%	45.00%	46.27%	0.062
	CAMPR3_DA	76.74%	2.78%	6.77%	−0.239
Fish	Our method	100.00%	97.00%	97.18%	0.810
	iAMPpred	91.30%	1.63%	6.92%	−0.117
	iAMP-2L	86.96%	99.46%	98.72%	0.882
	ADAM	95.65%	99.18%	98.97%	0.912
	DBAASP	82.61%	80.00%	81.58%	0.620
	MLAMP	91.30%	99.46%	98.97%	0.908
	CAMPR3_RF	91.30%	1.36%	6.67%	−0.130
	CAMPR3_SVM	95.65%	2.18%	7.69%	−0.034
	CAMPR3_ANN	82.61%	50.68%	52.56%	0.157
	CAMPR3_DA	86.96%	1.36%	6.41%	−0.194
Human	Our method	97.83%	92.17%	92.33%	0.482
	iAMPpred	91.30%	22.88%	24.73%	0.055
	iAMP-2L	54.35%	98.18%	96.99%	0.482
	ADAM	52.17%	98.91%	97.64%	0.534
	DBAASP	40.54%	86.84%	64.00%	0.310
	MLAMP	50.00%	98.36%	97.05%	0.464
	CAMPR3_RF	93.48%	0.85%	3.36%	−0.092
	CAMPR3_SVM	82.61%	1.09%	3.31%	−0.215
	CAMPR3_ANN	69.57%	48.67%	49.23%	0.059
	CAMPR3_DA	84.78%	1.46%	3.72%	−0.167
Insects	Our method	100.00%	97.56%	97.82%	0.900
	iAMPpred	94.44%	39.11%	45.04%	0.217
	iAMP-2L	94.44%	96.67%	96.43%	0.835
	ADAM	100.00%	96.67%	97.02%	0.870
	DBAASP	70.37%	90.91%	73.85%	0.469
	MLAMP	72.22%	98.00%	95.24%	0.740
	CAMPR3_RF	87.04%	1.33%	10.52%	−0.227
	CAMPR3_SVM	87.04%	1.33%	10.52%	−0.227
	CAMPR3_ANN	87.04%	43.33%	48.02%	0.192
	CAMPR3_DA	79.63%	1.56%	9.92%	−0.314
Mammals	Our method	92.79%	94.56%	94.46%	0.673
	iAMPpred	95.50%	68.94%	70.54%	0.322
	iAMP-2L	68.47%	98.73%	96.90%	0.712
	ADAM	65.77%	99.48%	97.45%	0.753
	DBAASP	45.88%	83.02%	60.14%	0.295
	MLAMP	51.35%	98.44%	95.60%	0.568
	CAMPR3_RF	93.69%	1.27%	6.85%	−0.096
	CAMPR3_SVM	92.79%	1.91%	7.39%	−0.085
	CAMPR3_ANN	78.38%	48.58%	50.38%	0.129
	CAMPR3_DA	88.29%	2.14%	7.34%	−0.140
Plants	Our method	97.78%	97.94%	97.93%	0.851
	iAMPpred	90.00%	0.81%	6.35%	−0.190
	iAMP-2L	77.78%	98.67%	97.38%	0.773
	ADAM	84.44%	98.67%	97.79%	0.815
	DBAASP	34.94%	88.46%	47.71%	0.219
	MLAMP	58.89%	98.82%	96.34%	0.654
	CAMPR3_RF	86.67%	0.59%	5.94%	−0.264
	CAMPR3_SVM	83.33%	0.88%	6.01%	−0.282
	CAMPR3_ANN	74.44%	47.57%	49.24%	0.107
	CAMPR3_DA	75.56%	1.10%	5.73%	−0.357

RF = random forest; DT = decision tree; KNN = K-nearest neighbor; SVM = support vector machine; ANN = artificial neural network; DA = discriminant analysis.

**Table A3:** Physicochemical properties and groupings of amino acids [13].

Physicochemical Properties	Group		
	Class 1	Class 2	Class 3
Charge	Positive K, R	Neutral A, N, C, Q, G, H, I, L, M, F, P, S, T, W, Y, V	Negative D, E
Hydrophobicity	Polar R, K, F, D, Q, N	Neutral G, A, S, T, P, H, Y	Hydrophobic C, L, V, I, M, F, W
Polarity	Polarity value 4.9~6.2 L, I, F, W, C, M, V, Y	Polarity value 8.0~9.2 P, A, T, G, S	Polarity value 10.4~13 H, Q, R, K, N, E, D
Polarizability	Polarizability value 0~0.108 G, A, S, D, T	Polarizability value 0.128~0.186 C, P, N, V, E, Q, I, L	Polarizability value 0.219~0.409 K, M, H, F, R, Y, W
Secondary Structure	Helix E, A, L, M, Q, K, R, H	Strand V, I, Y, C, W, F, T	Coil G, N, P, S, D
Normalized van der Waals volume	Volume range 0~2.78 G, A, S, T, P, D	Volume range 2.95~4.0 N, V, E, Q, I, L	Volume range 4.03~8.08 M, H, K, F, R, Y, W
Solvent accessibility	Buried A, L, F, C, G, I, V, W	Exposed R, K, Q, E, N, D	Intermediate M, P, S, T, H, Y
